# A case of steroid induced psychosis in a patient with mediastinal lymphoma

**DOI:** 10.1192/j.eurpsy.2024.658

**Published:** 2024-08-27

**Authors:** B. Orgaz Álvarez, M. Velasco Santos, P. Ibáñez Mendoza, Á. de Vicente Blanco, G. García Cepero

**Affiliations:** Hospital Universitario La Paz, Madrid, Spain

## Abstract

**Introduction:**

Corticosteroids are a key part of many cancer treatment regimens and neuropsychiatric side effects have long been recognised. Steroid-induced psychosis is a disorder classified under substance or medication-induced psychosis in the *Diagnostic and Statistical Manual of Mental Disorder, 5^th^ edition.* Management strategies include treatment with antipsychotic medication and reducing corticosteroid dosage.

**Objectives:**

To describe the case of steroid induced psychosis in a patient with mediastinal lymphoma and provide a concise literature review.

**Methods:**

Clinical case report and brief literature review.

**Results:**

27-year-old male with a diagnosis of Stage IV Primary Mediastinal Lymphoma according to the Ann Arbor classification was admitted to the Haematology ward for chemotherapy treatment (R-DA EPOCH). Two days after admission the patient developed acute psychotic symptoms consisting of thought block, kinaesthetic hallucinations, and delusions. Prior to admission, the patient had been on corticosteroid treatment for two months (up to 8mg/day of dexamethasone), with a significant dose increase (up to 200mg/day of prednisone) at the beginning of chemotherapy treatment two days prior to symptom development. The patient had no personal or family history of mental health issues, no substance misuse and had not received any psychopharmacological treatment prior to admission.

Medical evaluations including a cranial CT scan, an MRI, EEG, blood tests and lumber puncture were all within normal parameters, discounting organic or metastatic causes for the symptoms.

Considering a potential episode of steroid-induced psychosis, the patient was started on olanzapine at a dosage of 10mg per day. The patient exhibited a positive response, with symptoms alleviating within 24 hours of the initial dose. In terms of corticosteroid therapy, haematologists adjusted the prednisone regimen to 100mg per day, and due to the encouraging progress, the olanzapine dosage was subsequently reduced to 5mg per day.

**Conclusions:**

This case underscores the importance of considering the possibility of steroid induced psychosis as a differential diagnosis specially in patients on high dose steroids presenting with psychotic symptoms. A multidisciplinary approach is crucial to ensure optimum treatment and care.

**Disclosure of Interest:**

None Declared

